# Combination of Natural Compounds With Novel Non-thermal Technologies for Poultry Products: A Review

**DOI:** 10.3389/fnut.2021.628723

**Published:** 2021-06-08

**Authors:** Soukaina Barroug, Sonal Chaple, Paula Bourke

**Affiliations:** ^1^School of Biosystems and Food Engineering, University College Dublin, Dublin, Ireland; ^2^School of Biological Sciences, Institute Global Food Security, The Queens University Belfast, Belfast, United Kingdom

**Keywords:** poultry, non-thermal processing, natural compounds, *Campylobacter*, essential oils

## Abstract

Ensuring safe, fresh, and healthy food across the shelf life of a commodity is an ongoing challenge, with the driver to minimize chemical additives and their residues in the food processing chain. High-value fresh protein products such as poultry meat are very susceptible to spoilage due to oxidation and bacterial contamination. The combination of non-thermal processing interventions with nature-based alternatives is emerging as a useful tool for potential adoption for safe poultry meat products. Natural compounds are produced by living organisms that are extracted from nature and can be used as antioxidant, antimicrobial, and bioactive agents and are often employed for other existing purposes in food systems. Non-thermal technology interventions such as high-pressure processing, pulsed electric field, ultrasound, irradiation, and cold plasma technology are gaining increasing importance due to the advantages of retaining low temperatures, nutrition profiles, and short treatment times. The non-thermal unit process can act as an initial obstacle promoting the reduction of microflora, while natural compounds can provide an active obstacle either in addition to processing or during storage time to maintain quality and inhibit and control growth of residual contaminants. This review presents the application of natural compounds along with emerging non-thermal technologies to address risks in fresh poultry meat.

## Introduction

Fresh poultry meat and poultry products are highly perishable products but also have high potential as sources of human infection due to the presence and persistence of key pathogens in the poultry process chain. Outbreaks of foodborne illnesses in association with poultry products are one of the primary causes of outbreaks in the US and the EU. Among the reported numbers and notification rates of confirmed zoonoses in the EU in 2018, the top 5 are *campylobacteriosis* (246,158), salmonellosis (91,662), yersiniosis (6,823), and Shiga toxin-producing *Escherichia coli* (STEC) infections (6,073) (1, 2). Also, according to the Centers for Disease Control and Prevention (3), around 11, 2, and 1% of foodborne outbreaks are associated with chicken, turkey, and other poultry products, respectively, while turkey (609 illnesses) had the most outbreak-associated illnesses followed by chicken (487 illnesses). The pathogen *Campylobacter* caused up to 1.5 million illnesses each year in the US (4); thus, a focus on comprehensive and emerging methods for safety control in poultry processing is warranted.

**Figure 1:**
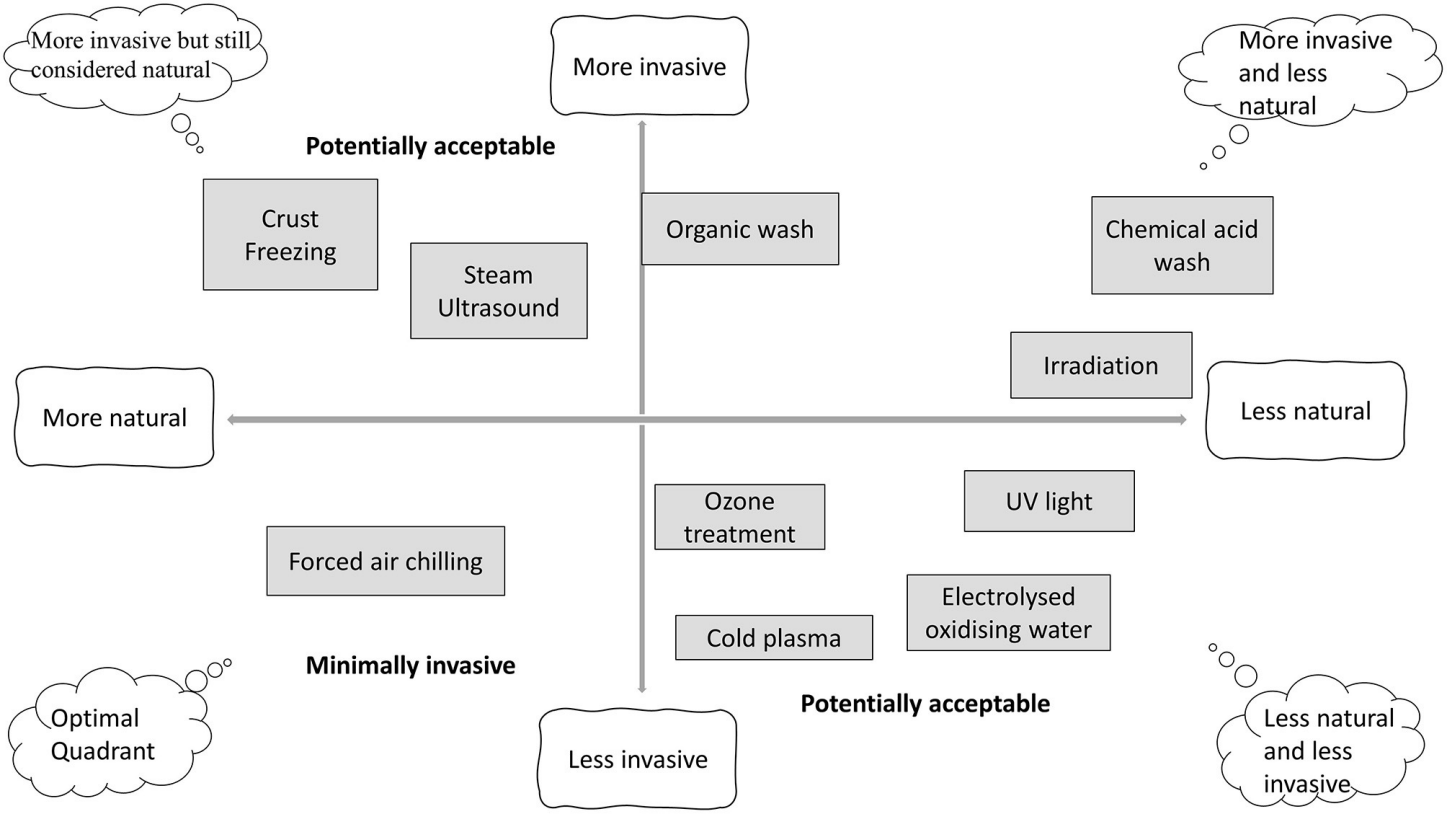
Schematics of perception analysis for different poultry decontamination treatments. Adapted from Safefood (5).

In order to provide safer poultry products, the food industry has developed and implemented preventive measures based on the Hazard Analysis and Critical Control Points (HACCP) and food safety management systems in combination with technological interventions, such as sanitization processes, refrigeration, and modified atmosphere packaging that can control identified potential microbial hazards during food processing and storage. However, according to the policy and consumer demand requiring sustainable, safe, and high-quality minimally processed foods, the food industry seeks alternative approaches to extend safe shelf life; such as irradiation, high-pressure processing (HPP), and natural green chemicals including bio-preservation or intelligent packaging. Figure 1 (6) illustrates the consumers' perception of 10 different meat decontamination processes based on how natural and invasive the process is considered.

The intensity of non-thermal processing treatment is vital for product safety and inactivation of pathogenic microbes. Han et al. (7) reported reactivation of sublethally injured microorganisms in a favorable environment during storage in meat and meat products. Spore-forming organisms such as *Clostridium botulinum* are resistant to HPP (8). The increase in a non-thermal process intensity to deal with recalcitrant microbiological issues may adversely affect foods' sensory properties (9). HPP can also alter the structure of polysaccharides and proteins, leading to textural changes in terms of hardness (10). Meat tenderness is the essential attribute that drives its consumer acceptability (11). Post slaughter, meat tenderization results from protease proteolysis of myofibrillar and cytoskeletal proteins as well as from the degradation of connective tissue substances, in particular collagen (12). Applying power ultrasound can produce free radicals, which imparts effective microbiological control, but which can also impact the product quality of high-fat foods due to oxidation (13). Similarly, irradiation can also cause undesirable organoleptic changes to high-fat foods and can induce color, odor, and taste effect on fresh meat products (14).

Thus, to overcome these shortcomings, there is potential to optimize non-thermal technology effects in combination with incorporation of natural compounds. Careful consideration of the mechanisms of action of individual non-thermal approaches may reveal what combinations can be successful across a range of food systems. For example, pulsed electric field (PEF) can cause cell membrane damage of microorganisms, enhancing sensitivity to antimicrobial agents like nisin (15). Similarly, application of naturally occurring antioxidant compounds such as rosemary extract, blueberry, or ascorbic acid can be used as an alternative for synthetic preservatives like butylated hydroxyanisole, butylated hydroxytoluene, and tertiary butylhydroquinone (16). Synergistic inactivation of microorganisms using non-thermal technology with natural compounds can be a promising way to increase the safety of poultry products while diminishing undesirable effects on some food characteristics. This review summarizes the key risks and published findings where non-thermal technologies have been combined with natural compounds and comments on the effects on the microbiological and physicochemical characteristics.

## Current Intervention Process Technologies Ensuring Poultry Microbiological Safety

**Figure 2:**
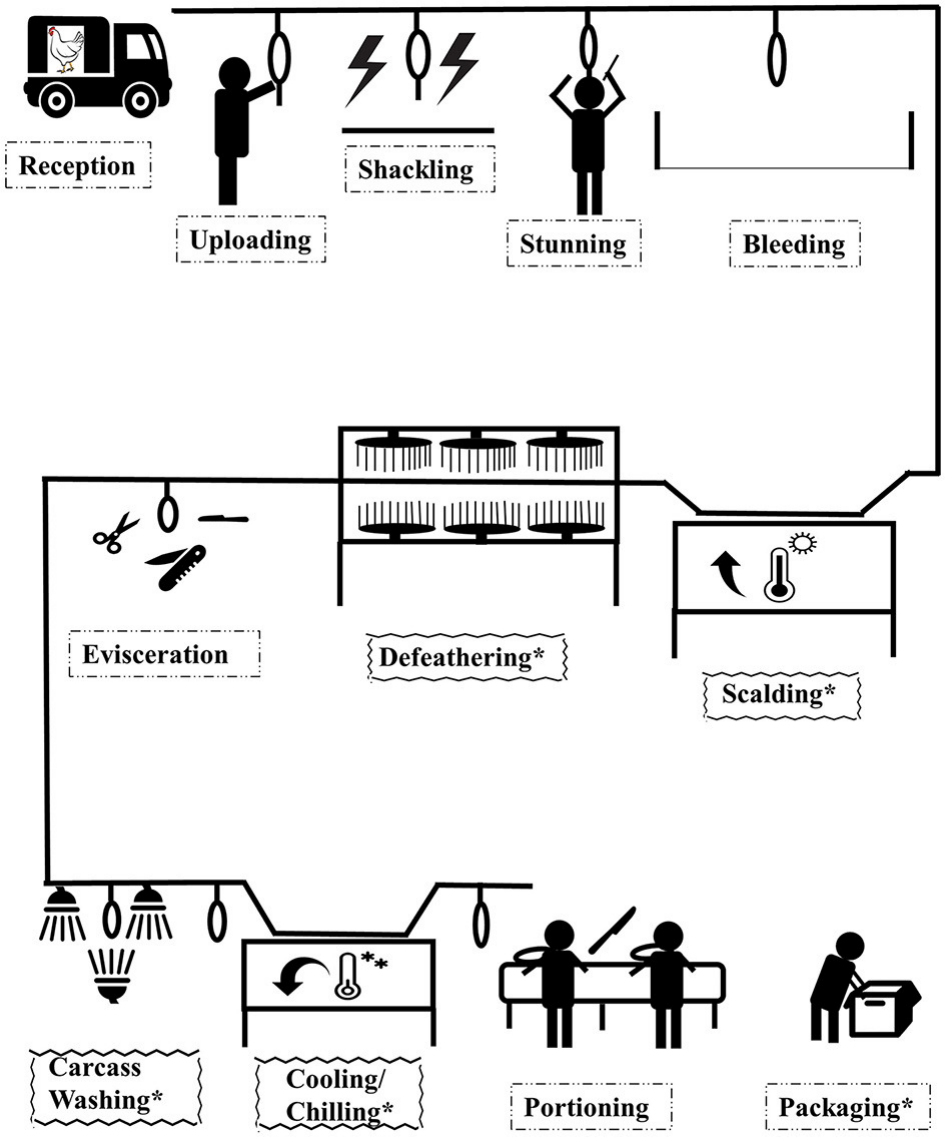
Overview of poultry processing commercial chain. “*” represents a post slaughter stage point where antimicrobial interventions can take place.

There are many approaches applied in poultry processing to maintain fresh poultry meat safety, which are generally classed as biological, physical, or chemical interventions (6). Electrolyzed water, hot water combined with rapid cooling, chilling and freezing (cold air and ice water), activated oxygen, and organic acids (lactic acid, oxidizing acids, and peroxyacetic acid) are examples of these decontamination processes currently used in poultry processing plants (17). The overview of the poultry processing plant is given in Figure 2. The proposed application of antimicrobial interventions in poultry processing can be implemented at carcass washing, scalding, defeathering, chilling/cooling, or packaging. The US Department of Agriculture (USDA) recommends the use of hot water above 74°C for sanitizing effect on carcasses.

High pressure, steam, and steam vacuum, as well as hot and cold water are some physical treatments commonly applied to meat carcass surface decontamination. Physical decontamination approaches currently used for chicken are steam and immersion in hot water (18). Due to the high temperature (100°C), microorganisms including natural microflora should be inactivated on the surface of the product within limited exposure times. Some limitations with stream usage are the deterioration of sensory characteristics attributed to changes of the color, and samples that look partially cooked, with shrunken skin (19). In steam vacuum processes, steam or hot water is sprayed on carcasses followed by vacuum treatment. This process is an effective method for spot decontamination at the slaughtering unit before the final chilling. The solutions of organic acids are frequently used in the chemical rinse to decontaminate the entire surface of carcasses. The most commonly used organic acids are acetic and lactic acids (20).

Peracetic acid, disodium phosphate, hexadecylpyridinium chloride, and sodium hypochlorite are the most utilized sanitizers during poultry processing (scalding and pre/post chilling) in poultry plants. Treatments with these antimicrobials can be online or off-line for reprocessing at different stages and temperatures and for different treatment times (21). Peracetic acid is an artificial disinfectant retaining good efficacy against poultry meat-related pathogens: *Salmonella* and *Campylobacter* (22). Sodium hypochlorite is commonly applied in water used for chilling/cleaning by spaying and/or immersion to reduce microbial load; however, the efficiency drops down significantly because of the interaction between organic matter in the meat and chlorine (23). Chlorine usage is prohibited in some countries including Germany, Denmark, and Belgium because of the potential interactions with organic matter within poultry carcasses, which can generate harmful chlorinated compounds (halo acetic acids, trihalomethanes, and chloramines) reported to be mutagenic and carcinogenic (23).

However, chlorine dioxide has also been used for sterilization, sanitization, and as disinfectant depending on its form (liquid or gas). Acidified sodium chlorite is an oxidative antimicrobial agent with a large activity spectrum (yeast, fungi, protozoa, viruses, pathogens, and molds). It is authorized by Food and Drug Administration (FDA) and Environmental Protection Agency (EPA) to be used in poultry (24). One of the best alternatives to classical sodium hypochlorite is electrolyzed water due to it low cost, high sterilization effect, and non-harmful effect (23). Specific organic acids can also be effective in terms of microbial inactivation and stability in the presence of organic mateial, namely, citric acid, lactic acid, succinic acid, and acetic acid. Nevertheless, some limitations are associated with the usage of such organic acids such as off-colors, odors, and flavors in addition to material corrosion (24).

## Microbiological Contaminants Of Concern In The Poultry Sector

### Microbial Spoilage

Owing to microbial spoilage, millions of pounds per annum of fresh poultry meat products are lost (25, 26). Poultry meat spoilage has dramatic effects by limiting shelf life (27) and negatively affecting the economy (28). The deterioration may result from quality and sensory damage to change in texture, odor, color, taste (29), and slime formation (27, 30). These changes are induced by enzymatic reactions, lipid oxidation (30), and action of the natural microflora within the poultry meat (28). Odor quality is affected by the production of volatile catabolites, while the deterioration in color happens throughout storage that is frequently related to biochemical reactions (between meat pigments, oxygen, and volatile microbial catabolites) and higher meat pH (30).

Several bacteria may be involved in poultry meat spoilage including coliforms, Enteriobacteriaceae, *Brochothrix thermosphacta, Pseudomonas, Aeromonas* sp., *Serratia*, lactic acid bacteria as *Lactobacillus oligofermentans, Leuconostoc gelidum* subsp. *gasicomitatum* (29), *Lactococcus, Vagococcus*, and *Carnobacterium* (27). The dominant spoilage bacteria is *Pseudomonas* spp. (26) due to its ability to assimilate, penetrate, and metabolize many meat compounds that other bacteria cannot use (30). When the total viable count achieves or exceeds 7 Log CFU/g, spoilage is deemed to ensue (29, 30).

### Pathogens

*Campylobacter, Salmonella, Listeria monocytogenes*, and *Staphylococcus aureus* are some of the primary pathogens naturally present or contaminants of poultry meat products (31, 32). *Clostridium perfringens, Listeria innocua*, and *Aeromonas* spp. were also identified in poultry meat (29). Recently, *Helicobacter pullorum* and *Acrobacter* gained considerable attention as relevant agents of poultry meat infections (33). *Staphylococcus saprophyticus* is another relevant agent of poultry product contamination, which varies from *S. aureus* because of its virulence factors and genetic profile (34). *C. perfringens* is widespread in the tract of birds (35), is responsible for enteric diseases in poultry (36), and is known for potential to generate extracellular enzymes and large numbers of toxins (37). At a certain microbial load, toxin production is upregulated (38). The enterotoxins delivered are associated with human gastrointestinal illnesses—enterotoxemia where toxins induce organ damage upon entering the circulation (36, 38).

### *Campylobacter* spp.

Poultry is the natural host/reservoir of *Campylobacter* spp. (39), which is present in the intestinal tract of birds, skin, and feathers (40). *Campylobacter jejuni* and *Campylobacter coli* are the main zoonotic enteropathogen causative serovars of human campylobacteriosis (41) and with high prevalence (42). *C. jejuni* is dominant by comparison with *C. coli* (39). At each point of the production chain, the proportions of these two isolates may be reversed by passing from one stage to the other. This could be attributable to the resistance or the susceptibility to a particular isolation technique introduced during the test of the collected samples and/or the feed withdrawal (41). They are microaerophilic and thermophilic (43) and require specific environmental conditions to develop (44).

It was reported that for 25.7% of chicken broiler carcasses tested, both liver (surface and internal tissue) and ceca were *Campylobacter* positive. However, for 83% (58/70) of carcasses tested, *Campylobacter* was isolated at least once in one of these compartments (42), and it is of note that the study pointed out that dissimilar subtypes of *Campylobacter* could simultaneously contaminate the same broiler carcasses.

### *Salmonella* spp.

*Salmonella* spp. is a facultative anaerobic Gram-negative genus (44), belonging to Enterobacteriaceae family. It is motile (except for *Salmonella enterica* Gallinarum and Pullorum) (45). It grows at optimum environmental conditions of pH 6.5–7 and temperature around 37°C (45). It is ubiquitous and able to survive in water for several months and in a dry environment for up to 2 weeks (46).

*Salmonella* is one of the main causative agents of foodborne disease globally (47). Animal-based foods such as beef, poultry, and pork are the major sources of salmonellosis (48), where human salmonellosis is mostly due to consumption of poultry products (44). *Salmonella* can persist throughout the processing chain from the farm to the fork (47). Enteritidis, Newport, and Typhimurium are serotypes commonly identified (49). The main genes encoding for the virulence are in both virulence-associated plasmid and pathogenicity islands. These genes are involved in internalization, epithelial cell invasion, survival, and replication, have a significant role in systemic infection (50), and may present a target for intervention technologies design.

## Natural Compound-Based Interventions Significant To Poultry Sector

### Spices and Herbs

These natural compounds are used in foods for flavoring and preservation and as additives but also for medicinal and therapeutic goals (anti-oxidative, immune modulators, anti-inflammatory, and antimutagenic). Their utilization in foods can have beneficial effects for shelf life extension as well as improvement of the organoleptic characteristics (51). The antioxidant potential of herbs or spices can prevent or decrease lipid oxidation (52) attributed to the action of phenolic compounds (53).

Clove and rosemary are two aromatic spices known for their antimicrobial and antioxidant potential. Antimicrobial activities of rosemary and clove extracts were tested separately or in combination against pathogenic and spoilage bacteria related to meat, namely, *L. monocytogenes, E. coli, Pseudomonas fluorescens*, and *Lactobacillus sake*, as well as against native microflora in poultry meat samples, where the combination of both provided enhanced microbial inactivation potential. The combination of the herb and spice also maintained or improved the sensory characteristics of fresh meat, reduced lipid oxidation, and extended shelf life up to 15 days (53).

Curcumin is a US FDA-approved safe plant pigment used for cooking, reputed for its health benefits. It acts as antioxidant, anti-inflammatory, and antiproliferative, but drawbacks for use in foods are related to the color, taste, and quality alterations (54). Corrêa et al. (54) analyzed the antimicrobial effect of two photonic approaches, ultraviolet (UV)-C and curcumin-mediated photodynamic inactivation (PDI), for control of *E. coli* and *S. aureus* inoculated on chicken breast cubes. Limitations were associated with these treatments, as the UV-C light or the curcumin-mediated PDI treatment with emission at 450 nm were not absorbed in all areas and did not significantly penetrate the chicken meat surface, with 1–2 log_10_ CFU/ml reductions observed (54).

### Essential Oils

Essential oils (EOs) are aromatic secondary metabolites and concentrated plant extracts. They can be obtained by steam distillation, expression or supercritical extraction with carbon dioxide from different parts of the plant, for example, bark, flower, fruits, seeds, leaves, or roots. Many EOs have found application in poultry feed as an alternative to antibiotics due to antioxidant, antiseptic, and insect repellent properties as well as immune-modulatory effects (55). EOs are chemically diverse compounds; hence, their antimicrobial activity varies from compound to compound. However, due to their hydrophobic nature, they are likely to enter cell membranes of microbes or eukaryotes (56). The main limitations of using EOs in food products are the strong flavor imparted on foods (56), the heat-labile nature, and volatile characteristics (57).

Rosemary extract is well-known for its antimicrobial activity, which is related to its phenolic composition (e.g., rosmarinic and carnisic acids). Inactivation of cellular enzymes was seen to result from the effect of phenolic compounds (16). Treatment of *Salmonella typhimurium* with thyme EO caused a rise in the electrical conductivity, which appears to result from the destruction of the cell membrane as well as electrolyte leakage. Additionally, quantitative analysis reported a significant drop in the protein contents, DNA, and ATP by 55.42, 54.03, and 52.64%, respectively, when compared to the control (57). Likewise, EOs (lemon oil, lemon grass oil, lime oil, garlic oil, onion oil, pimento berry oil, oregano oil, thyme oil, and rosemary oil) had higher antimicrobial potential against four *Campylobacter* strains when compared to organic acid (ascorbic acid, citric acid, and lactic acid). Oregano EOs specifically displayed the higher inactivation potential against *C. jejuni*, where the minimal inhibitory concentration was equivalent to 62.5 ppm (18).

The application of 0.1% oregano EO was effective for extending the shelf life up to 5–6 days for fresh chicken breast meat before packaging (58). The authors pipetted 0.1% of oregano EO in the low-density polyethylene (LDPE)/polyamide (PA)/LDPE barrier pouches, which was later subjected to either air or modified atmosphere packaging (MAP). The lipid peroxidation and deterioration of sarcoplasmic proteins were controlled to extend the shelf life of chicken breast up to 2 weeks at 4°C with the application of 0.5% of both thyme and *Melissa officinalis* balm EOs. These EOs were applied on the chicken breast slices by dipping method for 15 min. The results highlighted that thyme (0.5%) was more effective in inhibiting the growth of *E. coli*, whereas balm (0.5%) was more effective on the *Salmonella* spp. (59). The combined effect of ethylenediaminetetraacetic acid (EDTA) (1.5% w/w) lysozyme (1.5% w/w), rosemary oil (0.2% v/w), and oregano oil (0.2% v/w) was effective on extending the shelf life of vacuum-packed semi-cooked coated chicken filets stored at 4°C (60). EDTA and lysozyme were applied by spraying technique on the surface of the chicken surface, while rosemary and oregano oil were pipetted in the LDPE/PA/LDPE pouch barriers containing chicken samples.

### Organic Acids

Organic acids are naturally occurring compounds present in many foods and can be produced during the fermentation process. They are added in foods as acidulants, preservatives, or flavorants. The commonly used organic acids are lactic, acetic, malic, and ascorbic acid, etc. The mechanism of inactivation of these acids is through lowering of pH, pKa value along with penetration of undissociated compounds through the cell membrane and its dissociation inside the cell, thus affecting the bacterial membrane (61, 62). In poultry products, the salts of organic acids such as potassium or sodium lactate and sodium diacetate are used to inactivate *L. monocytogenes*, and buffered citrate is used to enhance flavor (63). The maximum level for potassium and sodium lactate is 4.8% by weight of total formulation in various meat and poultry products. For sodium diacetate, the maximum permitted level is 0.25% by weight of total formulation when used as either antimicrobial agent or flavoring agent (64).

## Non-Thermal Technologies And Their Combinations With Natural Compounds In Poultry Processing

**Figure 3:**
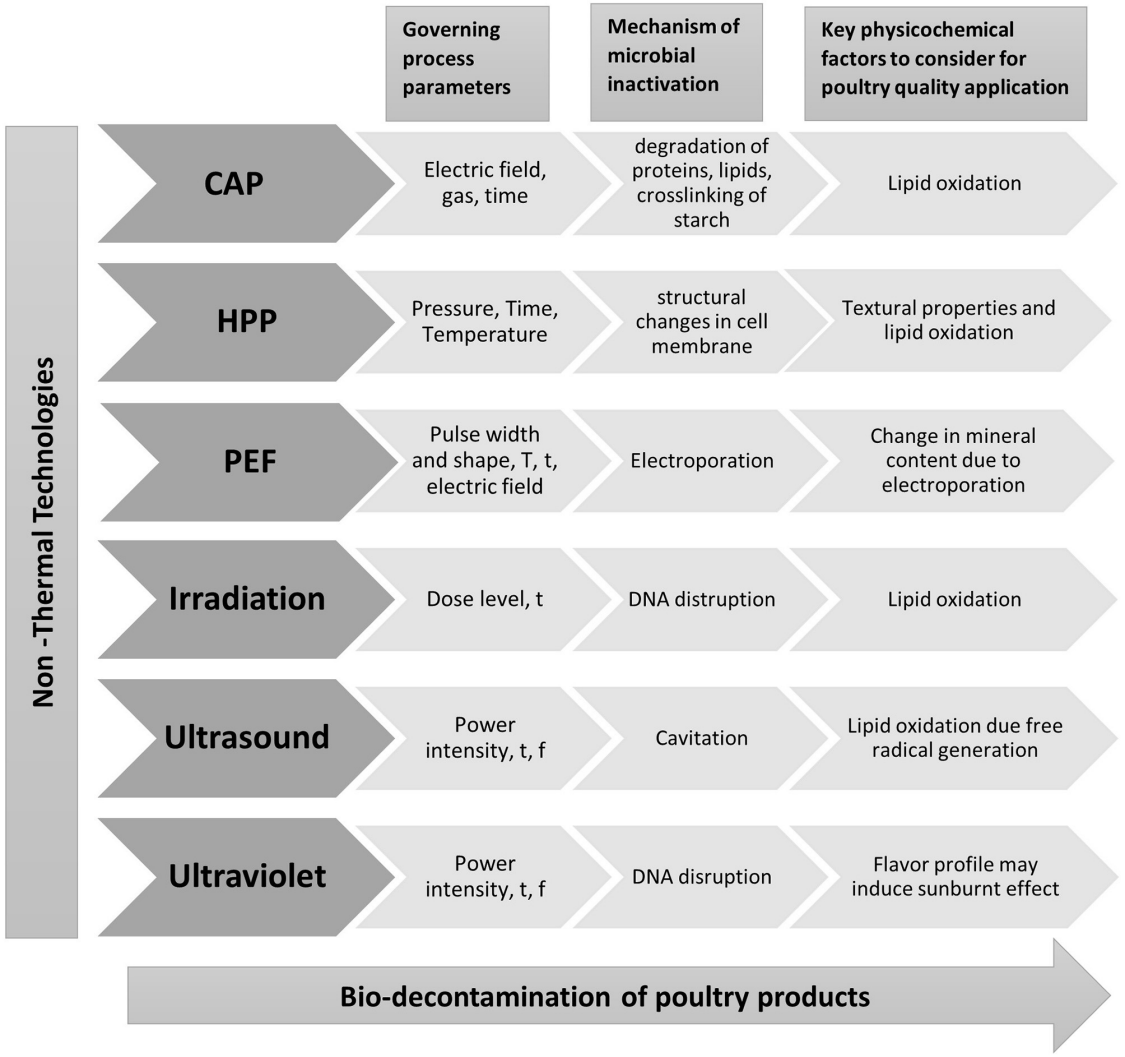
Illustration of key parameters of non-thermal technologies, their mechanism of action, and critical factors to consider for poultry application, where T represents temperature, t is time, and f is frequency. CAP, cold atmospheric plasma; HPP, high-pressure processing; PEF, pulsed electric field.

Non-thermal technologies, such as HPP, PEF, ultrasound, UV, irradiation, and cold atmospheric plasma (CAP) can retain nutritional as well as sensory properties of food in shorter treatment times and low operational temperatures (65). Extensive research on the application of various non-thermal technologies to poultry meat has been conducted in recent years. The effect of non-thermal technologies on microbial and physicochemical properties will be discussed in this section. The incorporation of natural compounds with non-thermal technologies can give an additional hurdle enhancing antimicrobial efficacy. Additionally, this will help adjust the processing conditions at lower intensity, giving improved physicochemical properties (66). Few studies have focused on combining natural compounds with the non-thermal treatment as summarized in Table 1 illustrating its impact on both physicochemical and microbial properties. A pictorial representation of governing processing parameters, mechanism for microbial decontamination, and key physicochemical parameters to take into consideration while application of the non-thermal technology in the poultry products is depicted in Figure 3, and it will be further discussed in coming sections.

**Table 1:** Summary of combination trials of different non-thermal technologies with natural compounds on poultry products.

**Non-thermal technology**	**Natural compounds**	**Poultry products**	**Chemical observations**	**Microbiological observations**	**References**
CAP	Rosemary extract	Poultry ground meats	NA	- Reduction of the bacterial functional diversity - The lowest Maximum Population Size (54.65, 95% confidence interval [CI95%] ranges, 54.03–55.16) and slowest growth rate (hour) (0.03258, CI95% ranges, 0.0179–0.04726) in day 0 - At day 5 of storage at 4°C, the maximum population sizes of treated samples were statistically not significant comparing to day 0	(67)
	Rosemary extract	Ground chicken patties	- Lower pH values for rosemary samples - a* value was significantly affected by rosemary addition - Addition of rosemary extract prevented lipid oxidation for CAP	Rosemary extract significantly reduced the total plate counts with and without cold plasma treatment	(16)
	Thyme oil (TO)/ Silk fibroin (SF) nanofiber	Chicken and duck meat	- Thyme oil release was enhanced due to surface modification of SF by plasma treatment - Higher overall acceptability of chicken meat treated with plasma treatment and combination of TO/SF nanofiber	- The population of *Salmonella* Typhimurium on treated chicken meat reached 1.15 and 1.96 log CFU/g when stored at respectively 4 and 25°C for 7 days after been wrapped with plasma-Thymol oil-Silk fibroin nanofibers. Identic effects seen with the dusk meat treated with the same process	(57)
	Essential oils: *Crocus sativus* L., *Allium sativum* L., and *Zataria multiflora Boiss*	Breast chicken fillets	Overall acceptability and no undesirable impacts on both flavour and odour	- Associating CP and essential oils treatments of breast chicken fillet infected by *S. aureus* and *E. coli* lead to significant microbial reductions by at least 3–4 logCFU/g. - A synergetic effect due to the combination of three different EOs (*Crocus sativus L., Allium sativum L., and Zataria multiflora Boiss*.) and CP treatment reaching microbial reductions to great extent. - After 14 days storage, 2–2.7 logCFU/g microbial inactivation reported comparing to 4.9 logCFU/g of samples treated with only EOs	(68)
HPP	Articoat-DLP (lactic acid, acetic acid and sodium diacetate- active compounds)	Chicken breast fillets	- Significant increase in *L*-value - TBARS value remained same during storage - Increase in pH due to HPP	- *Pseudomonas* spp., *B. thermosphacta, coliforms, E coli* inactivated below detection limit - LAB reformed after 7 days storage time	(69)
	Carvacrol	Turkey breast ham	- Higher TBARS value for pressurised samples - Carvacrol addition decreased TBARS value of samples	- Carvacrol+HPP extend the lag phase for *Listeria*- Reduced the growth rate of LAB spoilage groups	(70)
	Thymol	Ground chicken	NA	- addition of thymol impacted the HPP sensitivity for iPEC O157:H7 and UPEC	(71)
PEF	Oregano essential oils	Raw chicken	NA	- No significant inhibition of C, jejuni if only treatment with PEF (0.25–1 kV/cm) applied. - Sequential treatment of PEF with immersion for 20 min in oregano essential oil (15.625 ppm) were effective against *C. jejuni* 1146 DF with maximum reduction of 1.5 log CFU/g	(18)
Ultrasound	Lactic acid	Broiler drumstick skin	NA	Ultra-sonication alone and with 1% lactic acid did not significantly affect aerobic plate count	(72)
	Lactic acid	Poultry skin	NA	- *Pseudomonas* was most sensitive to lactic acid than other gram-negative bacteria - Degree of reduction of gram-negative bacteria was dependent on treatment time and liquid medium (water or lactic acid)	(73)
	Oregano essential oil	Chicken breast	NA	0.3% oregano oil and ultrasound showed better inactivation of lactic acid bacteria, mesophiles and anaerobic bacteria at day 0 and during 21 days of storage	(74)

### High-Pressure Processing

HPP is a non-thermal technology for the sterilization and preservation of food products in which the product is subjected to high pressure (300–600 MPa) with or without the combination of heat. With the application of HPP, covalent bonds in food matrices are not broken and the effect on the food characteristics is minimal. HPP is based on Le Chatelier's principle and the isostatic principle. Le Chatelier's principle states that “if a change in conditions is applied on a system in equilibrium, then the system will try to counteract that change and restore the equilibrium.” The isostatic principle states that food products are compressed by uniform pressure from every direction and then returned to their original shape when pressure is released. HPP is currently used for liquid and high-moisture solid products (75). The first commercialized HPP meat products available are sliced cooked ham and precooked meals containing poultry, pork, chorizo, and different sausages in Spanish market (76, 77). Further details about commercialized meat product of HPP are detailed elsewhere (78).

#### Effect of High-Pressure Processing on Microbial Decontamination of Poultry Products

Tracz et al. (2015) investigated the potential of HPP to destroy a mixed culture of three stains of *C. jejuni* inoculated in chicken breast under different pressure conditions (200, 300, and 400 MPa) and treatment times (5, 10, and 15 min), where D values were lowest at the highest pressure applied (79). According to the pressure applied, the temperature varies from 0 to 10°C. When the lowest pressure (200 MPa) was applied, *C. jejuni* exposed resistance and no significant reduction was achieved regardless of the duration of HPP treatment. Gram-negative bacteria are generally more susceptible to pressure compared to Gram-positive bacteria (79). Sheen et al. (2015) reported also that the inactivation of *Salmonella* spp. in ground chicken was dependent on both treatment time and pressure level applied (80). It was highlighted that even at high pressure (550 MPa), while the highest temperature reached was 28°C, *Salmonella* recovered and resuscitated over storage at 10°C to achieve ~6 Log CFU/g at day 9 of storage. Therefore, despite the mechanisms noted of surface structure damage or disintegration, internal cell compound disappearance, and appearance of internal voids in the cells, some cells survived the HPP 15-min treatment (80), pointing to a need for combination approaches. Argyri et al. (2018) HPP treatment at 500 MPa for 10 min at 18–20°C resulted in a significant reduction, below detection limit, of both the native microbiota of chicken and a cocktail culture of three different strains of *Salmonella* (31). Furthermore, *Salmonella enteritidis* inoculated on chicken at different initial concentration levels stayed below or just at detection limits during the storage at 4°C over 18 days (30). Working with a cocktail of *Listeria monocytogenes*, also Argyri et al. (2019) perceived the capability of HPP in maintaining safety and extending the shelf life of chicken (30, 32). Xu et al. (2020) reported D_10_ values for multi-isolated cocktails of extraintestinal pathogenic *E. coli* (ExPEC) to HPP (400 MPa, 0–25 min) on ground chicken, where 3.26 min was the average and the highest temperature value reached during the treatment was 25°C (81). Increasing the pressure to 600 MPa provided more than 6 log reduction within 3 min with no bacterial recovery after 4 min (81). The inactivation effect of HPP on two different strains of *E. coli* on ground chicken was assessed while the temperature remained under 40°C, where a significant resistance of uropathogenic *E. coli* (UPEC) by comparison with intestinal pathogenic *E. coli* (iPEC) O157:H7 at 450 and 500 MPa was reported (71). Liu et al. (2012) stated that *C. jejuni* HCJ2316 exhibited high resistance to pressure (2.8 Log CFU/g reduction), whereas the others were more susceptible to treatment and achieved 5 Log CFU/g reduction (82). However, the microbial recovery upon pressure treatment of *C. jejuni* is iron-dependent (82).

#### Effect of High-Pressure Processing on Physicochemical Properties of Poultry Products

Lipid oxidation is one of the major causes of deterioration of meat during storage. The chicken meat contains a higher amount of unsaturated fatty acids compared to other animal meats, which makes it more susceptible to lipid oxidation. The most common method to determine the lipid oxidation is Thiobarbituric acid reactive substances (TBARS) analysis (24). The lipid oxidation was particularly affected by working pressure; for low pressure (400 MPa and less), significantly less change in TBARS value was reported, while high pressure (500 MPa) have a higher impact on TBARS value (83). Similar effect was observed when chicken breast filets was treated with 450 and 600 MPa, while no significant change in TBARS value was observed at 300 MPa (84). The pressure of 800 MPa has the most detrimental on TABRS value (85).

#### Combined Effect of High-Pressure Processing and Natural Compounds on Poultry Products

A synergistic effect was recorded using nisin (200 ppm) and HPP (450 MPa) at 20°C, enhancing the microbial reduction of mechanically recovered poultry meat, specifically, the inactivation of both aerobic mesophile and psychrotroph populations was greater by comparison with HPP treatment on its own (86). Other researchers outlined the strong synergistic effect of combining hydrostatic pressure treatment (250 MPa for 30 min at 25°C) and 1% food additive (citric acid, nisin, and wasabi extract) in completely reducing the microbial concentration of *S. enteritidis* to undetectable levels (87). Combining HPP (300–400 MPa) with thymol (100–200 ppm) provided a large inactivation effect on separate cocktails of iPEC O157:H7 (0.94–5.16 Log CFU/g) and UPEC (0.41–4.66 Log CFU/g) in ground chicken samples (71).

### Pulsed Electric Field

PEF uses short pulses of high voltage (5–80 kV) for microbial inactivation. The food is placed between two electrodes, and an external electric field is applied, which induces the movement of ions along the direction of lines of force of the applied electric field inside as well as outside the cells. This causes the accumulation of ions on the membranes, causing polarization of the cell, which results in thickness reduction of the membranes due to the forces of attraction between oppositely charged ions on either side of the membrane (88). Because of the potential for cell membrane permeabilization, PEF is a promising technology to modify several qualities of meat, such as color, texture, and water-holding capacity, and enhance mass transfer during curing and brining. However, applications to date can be limited in solid products due to conductivity requirements (89).

#### Effect of Pulsed Electric Field on Microbial Decontamination of Poultry Products

The cell membrane is commonly referred to as the only target of PEF contributing to bacterial cell death (90). Treatments with PEF display reversible or irreversible damages on the cell membrane by disorganizing the structure, which yields the breakdown of the semipermeable barrier due to formation of pores in the membrane (18), leading to irreversible electro-permeabilization of the cell membrane; however, recovery can occur in optimal conditions (91). Process parameters of pulse frequency and strength of the electric field of PEF have been demonstrated to affect the microbial inactivation (92).

Reduction in population densities of *S. enteritidis* and *S. typhimurium* strains suspended in citrate-phosphate buffer was greater with increasing both treatment time and electric field above 9 kV/cm (93). It was demonstrated by Clemente et al. (18) that the treatment of chicken thighs with PEF did not result in any significant reduction of *C. jejuni*. PEF was not sufficient to reduce cell concentration of *S. enteritidis, E. coli*, and *C. jejuni* on raw chicken (92). However, this non-thermal technology is suggested to be suitable for treating process waters used in poultry processing as well as for poultry scald (92).

#### Effect of Pulsed Electric Field on Physicochemical Properties of Poultry Products

Several studies have reported the ability of PEF treatment to modify sensory characteristics, texture, and water-holding property of the meat products, further improving the mass transfer properties (94–96). Meat is considered an excellent source of minerals, such as zinc, iron, phosphorus, and calcium (97). PEF induces irreversible electroporation in meat and affects cellular permeability and mass transfer. Studies suggest that PEF-applied products have changes in mineral content when compared to control. Khan et al. (98) examined the effect of low (2.5 kV, 200 Hz) and high PEF (10 kV, 200 Hz) on four nutritionally important minerals (P, K, Fe, and Zn) of raw and cooked chicken breast. For raw chicken, non-significant changes in mineral content were noted; however, with cooking, a decrease in P, K, and Zn was observed and the concentration of Fe was not affected by treatment or cooking. In another study conducted by Khan et al. (99), the authors found higher concentrations of Ni and Cu for both low (2.5 kV, 200 Hz) and high PEF (10 kV, 200 Hz) than control. Thus, it is vital to study the migration of minerals into meat products due to PEF treatment and should be checked under regulatory limits.

#### Combined Effect of Pulsed Electric Field and Natural Compounds on Poultry Products

Recent work revealed that chicken oyster thigh artificially contaminated by *C. jejuni* 1,146 DF (final concentration 4.41 ± 0.20 log_10_ CFU/g) and treated with only PEF (0.25–1 kV/cm) did not demonstrate any significant inhibition potential. However, sequential treatment of PEF (1 kV/cm) and immersion in buffer with oregano EO (15.625 ppm) for 20 min resulted in a significant reduction close to 1.5 log_10_ CFU/g (18).

### Ultraviolet

UV light is electromagnetic radiation with wavelength from 10 to 400 nm. UV lights fall in the range between visible light and X-rays. To control surface contaminations on food products, UV-C light has received US FDA approval (Approval-2010). High-intensity pulsed UV light has been approved by FDA up to 12 J/cm^2^ (100). UV-C light can be used in Europe; however, in Germany, the use is limited to water, fruit, vegetables, and stored hard cheese (101). UV-C has a wavelength range of 220–300 nm (102) and is known for its antimicrobial effect (103), where the specific mechanisms of action include targeting of the nucleic acids (DNA, RNA) within the bacterial cell and generation of pyrimidine dimers (104). This latter results in the bonding of two adjacent pyrimidine bases, provoking obstruction of transcription and translation, respectively, and suspending vital cellular functions (102, 105, 106).

#### Effect of UV on Microbial Decontamination of Poultry Products

There are some limitations of using UV in poultry processing: UV-C light is not absorbed and cannot penetrate the chicken meat surface, which may affect microbial reduction. The antimicrobial efficacy of pulsed UV and UV-C has limitations also in terms of product density and treatment time (104, 107). The bactericidal effect of UV-C irradiation against *C. jejuni, L. monocytogenes*, and *S. typhimurium* on chicken breast was dose-dependent, where treatment at 5 kJ/m^2^ reduced *L. monocytogenes, C. jejuni*, and *S. typhimurium*, respectively by 1.29, 1.26, and 1.19 log cycles (102). Haughton et al. (105) examined UV effects against *S. enteritidis, E. coli*, and *Campylobacter* (*C. jejuni* and *C. coli*) when inoculated in liquid matrix, chicken skin and skinless chicken breast, food contact surfaces, as well as packaging materials. Treatment at a high dose equivalent to 0.192 J/cm^2^ provided complete microbial inactivation of *Campylobacter* strains suspended in a liquid matrix. By contrast, *Salmonella* and *E. coli* were more resistant to the similar UV dose (105). Food surface topography can shield microorganisms and limit UV treatment efficacy (104, 107). Isohanni and Lyhs (103) highlighted that although UV treatment was effective in reducing *C. jejuni* on surface medium by 6.3 log cycles per square centimeter. However, only 0.8 and 0.7 log cycles reduction were achieved on broiler skin and on broiler meat, respectively, with a dose of 32.9 mW/s per square centimeter. UV light seems to works well on smooth surfaces (108). Bacterial multilayer overloading as well as overlapping, and in the presence of cell, organic compounds protect to target bacteria from UV irradiation (104, 107).

#### Effect of UV on Physicochemical Properties of Poultry Products

UV light can form off-flavors due to the photochemical effect on the lipid fractions of product or due to absorption of ozone and oxides of nitrogen (101). This leads to the development of lipid peroxidation causing off-flavor. The hexanal aldehyde is a volatile secondary lipid oxidation product, and it is indicative of fatty aldehydes by headspace/gas chromatography–mass spectrometry (GC-MS). McLeod et al. (104) detected an increase in hexanal content of the raw chicken filets treated with 10.8 J/cm^2^ in air, which was noted by the sensory panel as a “sunburnt flavor” giving a low sensory score. Interestingly, when the same chicken samples were cooked, the sensory panel was unable to identify the difference, and it scored fairly with the untreated sample (104).

#### Combined Effect of UV and Natural Compounds on Poultry Products

The combination treatment of UV-C light and clove EO was assessed against poultry-related pathogen *S. typhimurium* biofilms, generated on stainless steel coupon surfaces. Treatment with 1.2 mg/ml of clove EO followed with UV-C (76.41 mJ/cm^2^) induced a synergistic effect and resulted in no surviving cells (6.8 log CFU/cm^2^) embedded within the biofilms. It was demonstrated that the contact with clove EO made the cell easily accessible by UV-C due to the morphological damage occurring: flatter structure (109).

### Ultrasound

Ultrasound as a non-thermal approach applies sound waves with higher frequency (above 20 kHz) than the normal human hearing. The ultrasound frequencies used in the food industry are classified into three categories based on the frequency-power ultrasound: low frequency, high power range (20–100 kHz) and large-amplitude waves where typical applications are within altering physicochemical properties or structure of foods. For low-intensity ultrasound, in the range of 100 kHz to 1 MKz, chemical reactions are activated, and free radicals can form like hydroxyl ions that can have antimicrobial properties. High-frequency ultrasound is usually used in the food processing and food safety industry. When the cavitation bubble breaks, it forms hydroxyl ions, which can have antimicrobial properties (110).

#### Effect of Ultrasound on Microbial Decontamination of Poultry Products

The mechanisms of action are connected to cavitation generation, which eventually disturbs the cell permeability as well as causing thinning (111) and damage on the bacterial membrane (112) and “localized heating” (73) that yields cell inactivation. The cell metabolism is disturbed due to ion penetration of the cell cytoplasm upon permeability disruption caused by pressure gradients of ultrasound. These mechanisms are the result of the collapse of cavitation bubbles during the acoustic cavitation (73). A further utility of ultrasound treatments is the “de-agglomeration of bacterial clusters” (73).

Apparent characteristics of target microorganism type, physiological state, and morphology determine the efficacy of ultrasound. The efficacy is also dependent on the surface of food matrix and temperature (111). Moreover, other parameters interfere with efficacy, such as frequency and sonication treatment time (73). The peptidoglycan in the cell membrane of Gram-positive could be a reason behind the resistance to ultrasound by these bacteria compared to Gram-negative (112), and the susceptibility to ultrasound treatment may vary between strains from the same type. The resistance of cells on plates during *in vitro* experiments to sonication by ultrasound was higher in contrast to the susceptibility of both *Campylobacter* and Enterobacteriaceae in raw poultry (112).

It was highlighted that chicken breast subjected to high-intensity ultrasound promoted the growth of mesophilic, psychrophilic, and lactic acid bacteria compared to untreated samples, possibly resulting from the release of nutrients (113). However, it was pointed out that the presence of *E. coli* was lower for samples subjected to longer treatment (30–50 min) compared to non-treated, whereas for *S. aureus*, it significantly decreased after 50 min. The microbial reduction in previously contaminated chicken wings depends on both treatment time (3–6 min) and sonication environment solution where the treatment was in (1% solution of lactic acid or sterile distilled water). The combination of lactic acid and sonication (40 kHz, 2.5 W/cm^2^) had a bactericidal effect on all the bacteria tested and was considered suitable for poultry carcass skin decontamination (73). However, combining ultrasound treatment (37 kHz, 380 W, 5 min) with 70% ethanol induced the highest microbial reduction from chicken skin for three types of attachment by *S. typhimurium* loosely, intermediately, and tightly attached by respectively 2.86, 2.49, and 1.63 log CFU/g (114).

#### Effect of Ultrasound on Physicochemical Properties of Poultry Products

Marinating is mostly used to increase meat tenderness, enhance flavor profile, reduce cooking time, and increase the shelf life of meat. Ultrasound (40 kHz, 22 W/cm^2^) increased the marination efficiency of chicken breast when treated with 15 and 20 min (115). A similar increase in marination efficiency, cooking yield, and tenderization was reported when broiler chicken was treated for 20 min and 18 h marination (91% water, 6% NaCl, 3% sodium tripolyphosphate) (116). A positive influence of ultrasound frequency (25, 45, and 130 kHz) and treatment time (1, 3, 6, 16, and 24 h) on marination efficiency was also reported for chicken breast, giving higher uptake of sodium chloride (117). Ultrasound improved the marination properties of meat by breaking the integrity of muscle cell or by enhancing the enzymatic reactions in cell (11, 111). Thus, ultrasound can be used as an alternative to standard marination techniques used in the industry.

#### Combined Effect of Ultrasound and Natural Compounds on Poultry Products

The exposure of broiler drumstick skin to ultrasonic energy in water and submerged in 1% lactic acid did not show consistent effect in terms of reducing aerobic plate counts. The irregular characteristics of broiler skin surface were proposed as the reason behind the lack of microbial reduction by protecting bacteria in the skin crevices and avoidance of the cavitation (72). In contrast, other work showed the decontamination efficacy of sonication (40 kHz) of chicken wing skin in 1% lactic acid aqueous solution, where the reduction of *E. coli, Proteus* sp., *Salmonella anatum*, and *P. fluorescens* inoculated on the surface of the chicken skin significantly increased with treatment time rising from 3 to 6 min. Except for *E. coli*, the microbial reduction was higher when sonication was performed in an aqueous solution of lactic acid instead of water. This was explained by the presence of ions penetrating the cytoplasm due to the action of gradient pressure yielding from ultrasound and the presence of free radicals received in sonochemical reactions (73). Combining high-intensity ultrasound with 0.3% oregano EO treatment was the most appropriate combination to achieve the best reduction of lactic acid bacteria (2.30 log_10_ CFU ml^−1^), mesophilic populations (3.36 log_10_ CFU ml^−1^), and anaerobic bacteria (3.11 log_10_ CFU ml^−1^) present in chicken breasts at day 0 of refrigeration. However, the treatment with ultrasound alone was ineffective to control microbial growth during chilled storage, where the release of nutrient was suggested as a reason permitting microbial growth (74).

### Cold Atmospheric Plasma

Plasma is a quasi-neutral ionized gas composed of ions, free electrons, atoms, and molecules in their ground as well as the excited state. Plasma can be generated using any kind of energy, which can ionize the gas, and mostly electric or electromagnetic source are used for generation of plasma species. Plasma can be classified as a thermal plasma or non-thermal plasma. Thermal or non-thermal plasma processes can be designed to be delivered in a format that is cold or near room temperature at the point of application, which is of value for retaining quality and nutritional characteristics while providing efficient bio-decontamination resulting from reactive oxygen or nitrogen species, charged particles, electric field, UV as components of diverse mechanisms of action (118).

#### Effect of Cold Plasma on Microbial Decontamination of Poultry Products

It is well-documented that cold plasma (CP) has a large potential for controlling microbial quality, extending shelf life, and avoiding post-processing contamination (25). It not only induces bacterial decontamination but also inactivates a broad spectrum of microorganisms including fungi, viruses, and spores (119). Various plasma compounds have critical interventions in the microbial decontamination process like NO_2_, NO, O, O_3_, OH, H_2_O_2_, UV photons, charged particles, and electric fields (120). Bacterial cell etching, erosion, morphological alteration, nucleic acid damaging, protein oxidation, and loss of cell viability are the mechanisms of CP to retain microbial safety in foods (121). However, different parameters and mechanisms interfere with the gravity of damage occurring. The antimicrobial effects of CP are a function of process: duration of treatment, gas mixture, mode of exposure (direct or indirect), power source intensity, as well as intrinsic characteristics of product: surface topology, nature of samples treated (liquid, solid, or semisolid), and characteristics of the target cell (122).

In-package CP treatment configuration enhanced the microbial safety, avoided postprocess contamination, and retained toxicological safety of ready-to-eat chicken products (123). Using the *Salmonella* mutagenicity assay, no genotoxicity was seen in plasma-treated chicken breast (120). Tulane virus (1.08 ± 0.15 log CFU/cube), indigenous mesophilic bacteria (0.70 ± 0.12 log CFU/cube), and *Salmonella* (1.45 ± 0.05 log CFU/cube) from chicken samples were significantly reduced upon CP treatment (24 kV for 3 min), where increasing voltage (from 22 to 24 kV) and treatment time had a positive impact on the microbiological quality (123). However, the majority of cells showed morphological changes (cell flattened and other distortions) at 2,000 Hz, whereas at 1,000 Hz, only cell clumps appeared, and other cells were hollowed out (124). *S. typhimurium, E. coli* O157: H7, and *L. monocytogenes* populations on chicken breast reduced from 5.48, 5.84, and 5.88 log CFU/g, respectively, at 0 min to 2.77, 3.11, and 3.74 log CFU/g at 10-min plasma exposure (120). Other studies highlight the potential of in-package dielectric barrier discharge (DBD) (70 kV) in controlling poultry-related pathogens, namely, *Salmonella* and *Campylobacter*, and inhibiting the growth of spoiling bacteria (psychrophiles) from chicken breast treated and stored (5 days/4°C) (23), where increasing CP treatment time to beyond 60 s improved microbial reduction of psychrophiles, while no significant effect was seen against foodborne pathogens. The *in situ* decontamination potential of plasma-activated water (PAW) against *P. fluorescens* ATCC13525 previously inoculated on chicken skin pieces was associated with the plasma process parameters of plasma discharge frequency and treatment time (124), where the concentration of plasma-generated reactive species of nitrite, nitrate, peroxide, hydroxyl, and ozone increased with the discharge frequency (124).

#### Effect of Cold Plasma on Physicochemical Properties of Poultry Products

The reaction of myoglobin with hydrogen peroxide may produce choleglobin-inducing discoloration of meat. An increase in both L^*^ and b^*^ value was observed when chicken breast was treated with flexible thin-layer DBD (123); in contrast, application of DBD applied on chicken breast (110 kV, 60 kHz) showed a decrease in L^*^ value mainly due to slime formation after 9 days of storage at 4°C (28). However, Zhuang et al. (25) did not report significant changes in L^*^, a^*^, and b value when chicken breast was treated with 70 kV. The reactive species generated from the plasma can induce lipid peroxidation. Zhuang et al. (2019), Lee et al. (2019), and Moutiq et al. (2020) reported no changes in lipid profile of chicken breast after CP treatment, attributed to plasma reactive species being less damaging on chicken breast as compared to red meat due to variation in fat content (25, 28, 120, 123).

#### Combined Effect of Cold Plasma and Natural Compounds on Poultry Products

Yeh et al. (2019) considered the combined treatment effect of rosemary (*Rosmarinus officinalis*) extract (1%) and in-package DBD-CP on poultry ground meat (Table 1) (67). The treatment induced a reduction of the diversity in bacterial communities, and by day 5 of storage at 4°C, the maximum population densities of treated samples were similar to those at day 0 (67). Rosemary extract had a significant effect in reducing the total microbial counts not only of previously plasma-treated chicken patties but also of non-plasma-treated chicken patties (16). Thyme oil/silk fibroin nanofibers treated with CP were proposed as an active packaging approach with antimicrobial potential against *S. typhimurium* inoculated on poultry meat (chicken meat and duck meat). The inhibition potential of thyme EO/silk fibroin nanofibers treated with CP was reported as higher compared to thyme EO/silk fibroin nanofibers, with an increase in the rate of thyme oil released (23.5–25%) upon plasma treatment clearly noted (57). Sahebkar et al. (2020) found that associating CP (10 min at 32 kHz) and EO (in marinade solutions) treatments of breast chicken filet inoculated with *S. aureus* and *E. coli* challenge populations leads to significant microbial reductions by up to 3–4 log CFU/g (68). A synergetic effect was identified by combining three different EOs (*Crocus sativus* L., *Allium sativum* L., and *Zataria multiflora* Boiss.) and CP treatment, where the advantage of combining the EOs with CP was retained after 14 days of storage (68).

## Future Challenges For The Application Of Non-Thermal Technologies To Poultry Processing

In recent years, there has been intensive research and development of non-thermal process technologies for application in fresh food processing. EU regulations refer to “fresh meat” as meat that has not undergone any preserving process other than chilling, freezing, or quick-freezing, including meat that is vacuum-wrapped or wrapped in a controlled atmosphere (125). The application of a novel non-thermal technology will be subject to these rules, which may on the one hand lead to consumer skepticism toward acceptance of these technologies while on the other hand limit adoption of processes that can enhance safe and sustainable processing of food resources. To date, HPP and UV (with limitations in some countries) are approved. Thus, combining these approaches with other generally recognized safe approaches including approved natural compounds provides technical options to enhance poultry processing outcomes. The morphological characteristics of poultry products and the skin make the application of non-thermal technology very critical. For example, from a microbial safety perspective, the successful application of UV in poultry processing must consider density, and effects may be limited due to non-absorption and non-penetration of light in the chicken meat surface (104, 107).

While many plant EOs are considered generally safe by FDA, with increasing use, the daily dose intake remains a safety question (126). The adoption of EOs is controlled by a nexus of dosage level, antimicrobial efficacy (127), and the effect of organoleptic characteristics on consumer acceptability (128). The stability, strong smell, volatility, and limited solubility are technical issues to be considered from an efficacy perspective. These should also be considered in tandem with other processing features as the process or environment may stimulate the degradability of these compounds (129).

## Conclusion

Effective interventions, based on the combinations of emerging process technologies with the well-understood efficacies of nature-based compounds, can be designed to enhance safety and quality and minimize food loss in poultry processing. Studies to date have demonstrated that the order or sequence of application can be variable to address the key risks at different process stages, providing great flexibility if being considered as effective replacements for thermal or conventional chemical strategies.

## Author Contributions

All authors listed have made a substantial, direct and intellectual contribution to the work, and approved it for publication.

## Conflict of Interest

The authors declare that the research was conducted in the absence of any commercial or financial relationships that could be construed as a potential conflict of interest.
